# The role of the microbiome in gastrointestinal inflammation

**DOI:** 10.1042/BSR20203850

**Published:** 2021-06-11

**Authors:** David J. Sanders, Saskia Inniss, Gregory Sebepos-Rogers, Farooq Z. Rahman, Andrew M. Smith

**Affiliations:** 1 Department of Microbial Diseases, UCL Eastman Dental Institute, Royal Free Campus, University College London, London, United Kingdom; 2 Department of Gastroenterology, University College London Hospitals NHS Foundation Trust, 250 Euston Road, London NW1 2PG, United Kingdom

**Keywords:** Dysbiosis, Gastrointestinal physiology, Homeostasis, Inflammation, Microbiome, Mucosa

## Abstract

The microbiome plays an important role in maintaining human health. Despite multiple factors being attributed to the shaping of the human microbiome, extrinsic factors such diet and use of medications including antibiotics appear to dominate. Mucosal surfaces, particularly in the gut, are highly adapted to be able to tolerate a large population of microorganisms whilst still being able to produce a rapid and effective immune response against infection. The intestinal microbiome is not functionally independent from the host mucosa and can, through presentation of microbe-associated molecular patterns (MAMPs) and generation of microbe-derived metabolites, fundamentally influence mucosal barrier integrity and modulate host immunity. In a healthy gut there is an abundance of beneficial bacteria that help to preserve intestinal homoeostasis, promote protective immune responses, and limit excessive inflammation. The importance of the microbiome is further highlighted during dysbiosis where a loss of this finely balanced microbial population can lead to mucosal barrier dysfunction, aberrant immune responses, and chronic inflammation that increases the risk of disease development. Improvements in our understanding of the microbiome are providing opportunities to harness members of a healthy microbiota to help reverse dysbiosis, reduce inflammation, and ultimately prevent disease progression.

## Introduction

The human body is inhabited by a highly diverse population of microorganisms (microbiota) that has co-evolved with their human hosts over many millennia [1]. The human microbiome, a term more precisely used to describe the genomes of these microorganisms [2], is predominantly made up of bacteria [3], however archaea, viruses, and single-cell eukaryotes (e.g. fungi and protists) are also present [4–7]. These microorganisms are at least as abundant as the number of human host cells [3,8] and combined contain far more genes than the entire human genome [9]. Over the past few decades, research related to the microbiome has intensified, facilitated by rapid advances in culture-independent, high-throughput genomic and metabolomic techniques [10–12]. Consequently, a greater understanding of microbiota population composition and host–microbe interactions has been achieved, especially in the context of human health and disease [11,13,14]. Whereas a balanced microbiota has been shown to play an important role in the maintenance of human health, impairment or imbalance in the makeup of the human microbiota (dysbiosis) can disrupt homoeostasis and lead to the onset or exacerbation of human disease [15]. Multiple factors are known to influence the microbiota, however, studies have shown that the microbiome is more strongly influenced by an individual’s environment [16,17]. There are significant similarities in microbiota composition of genetically unrelated individuals who share a household, with approximately 20% of interperson microbiota variability associated with environmental factors such as diet, lifestyle, and medication [16].

The human microbiome can be separated into compartment-specific ecosystems that exist on the skin and along mucosal surfaces such as those of the oral cavity, gastrointestinal tract, lungs, and genitourinary system [1]. The largest concentration and diversity of microbiota can be found within the gut especially in the colon [1]. The mucosa, which consists of a single cell thick epithelium overlaying a layer of connective tissue called the lamina propria, provides the interface between the host and the environment and is equipped with specialised features, particularly along its apical surface, to allow physiological function whilst also being in contact with the microbiota [18]. The microbiota is however not functionally independent from the host mucosa and can fundamentally influence mucosal integrity, modulating host immune responses and mucosal inflammation.

Here we review the relationship between the microbiota and the mucosa, especially in relation to gut homoeostasis and mucosal inflammation. We first discuss factors that shape an individual’s microbiome and the impact the microbiome has on the intestinal mucosa during homoeostasis. We then explore how dysbiosis of the microbiome can lead to mucosal inflammation, resulting in the development of human disease, and highlight current and emerging therapies being used to suppress mucosal inflammation through targeting of the microbiome.

## Factors shaping the microbiome

**Figure 1:**
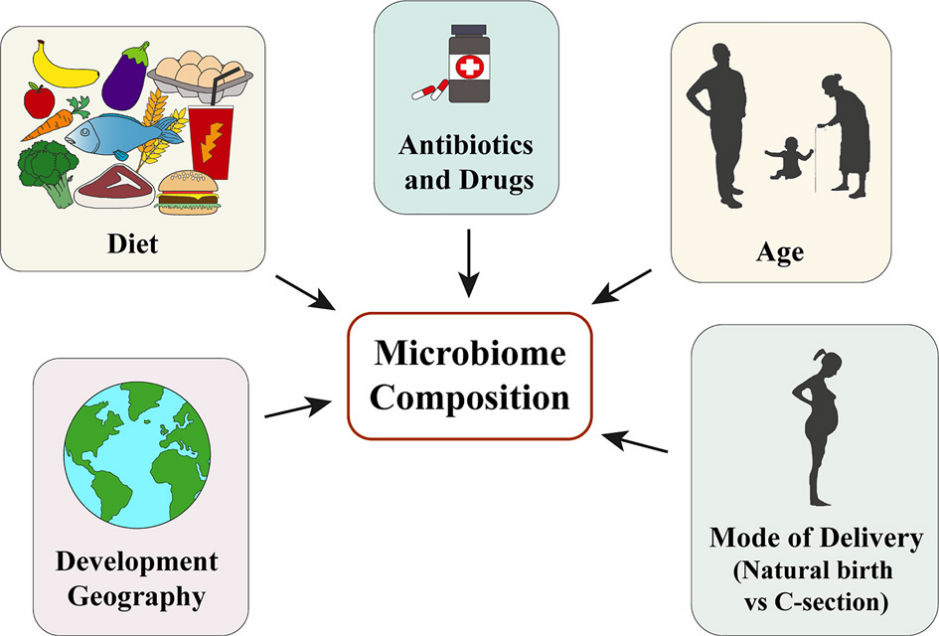
Factors that contribute to the shaping of the human microbiome

There is increasing evidence to suggest that there is a core microbiome shared between all individuals [19]. However, the composition and diversity of much of the gut microbiome varies greatly from person to person, adapting to both intrinsic and environmental factors [20,21]. Research to date has shown that environmental factors, mainly diet and medication, dominate over intrinsic factors, such as host genetics, in shaping the microbiome [16,22]. Age [23,24], geography [25], and birthing practices [26,27] are also known to be particularly important for determining microbiome composition (Figure 1).

### Diet

In the first year of life, the gut microbiome is relatively unstable becoming progressively more stable following weaning, taking on an adult form typically around 3 years of age [28]. Infant feeding practices as well as adult habitual diet play an important role in shaping the gut microbiome [29]. Studies looking into the effect of diet on the make-up of the intestinal microbiota have to date mainly focused on the so-called ‘Western’ diet, which is characterised by high levels of fat, sugar and refined protein [30,31], and diets that are high in fibre and low in red meat, such as the Mediterranean diet [24,32].

Differences in gut microbiome composition prior to weaning have been observed between breastfed and formula-fed infants. Breastfed infants have a microbiome dominated by Lactobacilli and *Prevotella*, whereas formula-fed infants exhibit a more diverse microbial population, dominated by Enterococci, Enterobacteria, Bacteroides, Clostridia, and Streptococci [33,34]. Breastmilk contains oligosaccharides which promote the growth of beneficial *Bifidobacteria* [35]. Bifidobacteria play a major role in the fermentation and conversion of oligosaccharides into short-chain fatty acids (SCFAs), such as butyrate and propionate, which promote healthy immune function (reviewed in detail below ‘The microbiome and intestinal homeostasis’) [36]. In addition to providing critical nutrients and bioactive compounds, human breast milk also plays an important role in the seeding of an infant’s gut microbiome, containing a variety of beneficial bacteria, including Lactobacilli and Bifidobacteria [37]. After weaning, the microbiota becomes more diverse and is dominated by Bacteroidetes and Firmicutes [38].

Studies looking at the adult gut microbiome have found that individuals consuming a Western diet experience a decrease in the total number of gut bacteria, particularly Bifidobacteria and Eubacteria, and an increase in pro-inflammatory bacteria-derived compounds [39–41]. A key aspect of the Western diet is a high intake of saturated fatty acids which has been linked to both a decrease in Gram-negative bacteria within the gut, particularly Bacteroidetes, and an increase in Lactococci [42,43]. Whilst there is currently a lack of consensus as to the precise effect of these dietary components on the microbiome, most studies have observed an overall decrease in bacterial diversity, a decrease in SCFA production, and an increase in harmful bacterial strains, such as pathogenic *Escherichia coli* (*E. coli*) [44,45]. In contrast with a Western diet, adults who consume a Mediterranean diet exhibit increased levels of Bifidobacteria, Lactobacilli, Eubacteria, and Bacteroides [46,47]. Furthermore, individuals who consume a Mediterranean diet have been shown to have increased levels of SCFA-producing bacteria, such as *Provetella* [48]. In addition to habitual diet, research has shown that dietary diversity, meal timing as well as short- and long-term dietary modifications can change the composition and activity of the adult gut microbiome [49–52]. Caloric restriction, for example, which is a nutritional intervention of reduced energy intake, has a strong influence on the gut microbiota [53,54]. It has been found that caloric restriction can slow down age-related decline in the microbiome, increase both microbial diversity and Bacteroidetes/Firmicutes ratio, as well as change host microbial co-metabolites leading to a decrease in host lipid biosynthesis and an increase in fatty acid catabolism [55,56].

### Antibiotics and drugs

Antibiotics are medicines used in the treatment of bacterial infections. Whilst they have proved to be an effective treatment against many bacterial diseases, their antimicrobial action profoundly affects the composition and function of the gut microbiome, causing dysbiosis by killing both pathological and beneficial bacteria, and allowing the expansion of resistant microbes [57]. The effects of antibiotics on the gut microbiome are potentially long-lasting, and their use in early life has been associated with an increased risk of developing several conditions including inflammatory bowel disease (IBD) and asthma [58,59].

Antibiotics can drastically reduce, or even fully eliminate, beneficial anaerobic bacterial species such as Bifidobacteria, Lactobacilli, Bacteroides, and Clostridia [60]. After only 7 days of antibiotic treatment, microbial diversity has been found to decrease by 25%, with core phylogenetic microbiota reducing from 29 to 12 taxa and antibiotic-resistant Bacteroidetes increasing 2.5-fold [61]. Consequently, antibiotic use can also result in reduced SCFA production [62]. The effects of antibiotics on the microbiome are however dependent on the type of antibiotic used. Clindamycin, which is a broad-spectrum antibiotic, can cause microbial changes that last for up to 2 years with no recovery in Bacteroides diversity [63]. Clarithromycin and Ciprofloxacin, which are used against *Helicobacter pylori*, are associated with a decrease in Actinobacteria and Ruminococci, respectively [64,65]. Vancomycin, which is used against *Clostridium difficile (C. difficile)*, causes an increase in Proteobacteria species and a decrease in Bacteroidetes, Ruminoccoci and Faecalibacteria levels, which can lead to both recurrent *C. difficile* infection (rCDI) and the growth of unwanted bacterial species, such as pathogenic *E. coli* [66,67].

Non-antibiotic drugs are also known to influence the composition and stability of the microbiome. A recent meta-analysis revealed that in addition to antibiotics, proton pump inhibitors (PPIs), metformin, and laxatives exhibit the greatest effects on gut microbiome composition and function [68]. PPIs reduce microbial diversity and cause taxonomical changes in the gut. Metformin significantly increases *E. coli* abundance and effects the number of SCFA-producing bacteria [68,69].

### Birth mode of delivery

Studies have shown that whereas vaginally delivered babies have a microbiome dominated by Lactobacilli and Prevotella, babies born by caesarean section (C-section) carry a microbiome dominated by Streptococci, Corynebacteria, and Propionibacteria [70,71]. Furthermore, babies born by C-section have been shown to have an abundance of potentially pro-inflammatory *Klebsiella* and *Enterococcus* bacteria [26]. A recent study reported that the abundance of *Klebsiella* and *Enterococcus* species in C-section born children at 1 week of life was associated with an increased number of respiratory infections over the first year [26]. Additionally, babies delivered by C-section have been shown to have lower total gut microbial diversity, delayed Bacteroidetes colonisation, and a subsequent immune system imbalance during the first 2 years of life which may result in the development of allergies [72,73].

### Age

Many studies have observed age-related changes to the gut microbiome. In infancy, the developing gut microbiome undergoes three distinct phases of progression: a developmental phase (months 3–14), a transitional phase (months 15–30), and a stable phase (months 31–46) [74]. Children and young adults have a higher abundance of Bifidobacteria and Clostridia, and a lower microbial diversity compared with adults [75]. In general, healthy adults exhibit high levels of Bacteroidetes and Firmicutes, and low levels of Proteobacteria, Actinobacteria, Fusobacteria, and Verrucomicrobia [20,76,77]. Throughout life, intestinal levels of Firmicutes decrease whilst Bacteroidetes levels increase. Elderly people have a gut microbiome enriched with Bacteroidetes and Proteobacteria and depleted levels of Bifidobacteria and Lactobacilli [24,78]. The transition from healthy adult to healthy old age is characterised by a decrease in microbial diversity, as well as an accumulation of potentially pro-inflammatory microbes and decrease in beneficial microbes [79].

### Development geography

To date, most studies investigating the link between the microbiome and geography have focused on differences in microbiome composition amongst three contrasting human populations: hunter gatherers, traditional farming or fishing communities, and Western industrialised communities [80–84]. When comparing the microbiomes of hunter gatherers with those of more developed communities, hunter gatherers were found to have a higher microbial diversity, with enrichment of Prevotella, Treponema, and Bacteroidetes [80,81]. In contrast, Western industrialised communities have higher levels of Bacteroides and Firmicutes, with an overall lower microbial diversity. Some studies suggest that the microbiomes of traditional farming and fishing communities exhibit an intermediate state between hunter gatherers and Western industrialised communities [82,85]. Factors thought to influence gut microbiome composition amongst hunter gatherers include a diet consisting of predominately starchy foods, limited access to modern medicine, and exposure to a wide variety of pathogens and parasites [82,83]. Traditional farming or fishing communities are thought to possess microbiomes with a relatively high taxonomic diversity, allowing the host to withstand pathogens and parasites, as well as to be able to respond to dietary fluctuations due to crop seasonality [83]. In Western industrialised societies, the gut microbiome is thought to be largely determined by diets high in refined protein and fat, good sanitation and hygiene practices, and the habitual use of antibiotics and other medications [80,81,84]. Some studies have also proposed that the lower microbiome diversity found in Western industrialised communities can be attributed to an overall loss of biodiversity due to industrialisation, pollution, and use of chemicals [86,87]. Furthermore, differences in sanitised drinking water may also have an effect on the composition of the gut microbiome [88,89].

## The microbiome and intestinal homoeostasis

The intestinal mucosa is highly adapted to be able to tolerate a large population of microorganisms and dietary antigens whilst preserving nutrient uptake and raising an effective immune response to pathogenic infection or commensal intrusion into the underlying host tissue [90]. For the most part, the microbiota maintains symbiosis with the gut environment forming a mutually beneficial relationship with the host. The gut provides a nutrient-rich habitat for the microbiota whilst the microbiota stimulates the host’s immune system, aids digestion, and provides otherwise unobtainable metabolites. In a normal healthy gut, the microbiota is diverse with an abundance of beneficial bacteria that help to maintain gut homoeostasis, promoting protective intestinal immune responses at the mucosal surface, and limiting excessive mucosal inflammation [91].

The microbiota can communicate directly with the host through host recognition of highly conserved structural components, termed microbe-associated molecular patterns (MAMPs) [92], such as lipopolysaccharides (LPSs), peptidoglycan (PGN), and flagellin. Recognition of MAMPs are achieved primarily through binding to pattern-recognition receptors (PRRs) expressed by intestinal epithelial cells (IECs) and immune cells. PPRs are a diverse family of transmembrane and cytoplasmic innate immune receptors, that include Toll-like receptors (TLRs) and nucleotide-binding oligomerisation domain (NOD)-like receptors (NLRs) [93]. PPR stimulation triggers intracellular signalling cascades leading to the expression of a range of immunomodulatory molecules that orchestrate early immune responses resulting in mucosal inflammation and further activation of innate and adaptive immune processes [94]. Whereas activation of PRR by pathogens and pathobionts is known to initiate pro-inflammatory signalling cascades that lead to mucosal inflammation, the commensal microbiota can use similar mechanisms to dampen inflammation and promote intestinal homoeostasis [95]. For example, polysaccharide A (PSA) from the ubiquitous gut commensal *Bacteroides fragilis* (*B. fragilis*) is recognised by the TLR1/TLR2 heterodimer, in co-operation with the C-type lectin PRR Dectin-1, triggering a signalling cascade through the phosphoinositide 3-kinase (PI3K) pathway to promote 3′,5′-cyclic adenosine monophosphate (cAMP) response element-binding protein (CREB)-dependent transcription of anti-inflammatory genes [96]. NOD2 stimulation by muramyl-dipeptide (MDP), a PGN motif, triggers intestinal leucine-rich repeat-containing G-protein coupled receptor 5 (Lgr5)^+^ stem cell survival and epithelial regeneration [97]. In addition to microbe specific constituents, there are also numerous microbiota-derived metabolites, such as SCFA, that stimulate a range of signalling pathways to further regulate mucosal immune responses and aid microbial symbiosis/tolerance [98].

### Direct microbial maintenance of intestinal barrier integrity

The intestinal mucosa forms physical, biochemical, and immunological barriers which allow for the symbiotic microbiota–host relationship to be maintained, controlling the microbial population, and reducing direct contact with the host [99]. Maintenance of these barriers are essential for preventing microbial invasion, excessive immune responses, and mucosal inflammation. As well as defending against pathogens through competition for nutrients and production of antimicrobial molecules [100,101], the gut microbiota also plays an active role in the maintenance of host mucosal barriers, which further prevents colonisation by opportunistic pathogens, limiting excessive mucosal inflammation, and preserving gut homoeostasis [99,100].

The physical barrier consists of a wall of IECs that are held together by cell junctions, particularly tight junctions (TJs), allowing only selective paracellular transport of water, ions, solutes, and some nutrients, preventing passage of microorganisms [102]. A mucus layer, predominantly formed of highly glycosylated mucins secreted by goblet cells, covers IECs and further contributes to the physical barrier preventing bacteria from interacting directly with host tissue [103]. The mucus layer also provides moisture and lubrication to protect IECs from dehydration and mechanical stress caused by the passage of food and peristaltic forces [104]. The small intestine contains one layer of mucus whereas the colon contains two: a loose outer layer that is permeable to bacteria and a dense inner layer that is impermeable and devoid of bacteria [105]. In the small intestine particularly, secretory molecules such as antimicrobial peptides (AMPs) and immunoglobulin (Ig)A are released and concentrated in the mucus layer, which further aid separation of the microbiota from the host mucosa [101,106]. In addition to targeting microbes directly and sequestering key nutrients to control microbiota biodiversity, these barriers can also modulate the host’s innate and adaptive immune responses [107,108] and drive up-regulation of mucin and TJ protein expression in IECs to maintain intestinal barrier integrity [109,110].

**Figure 2:**
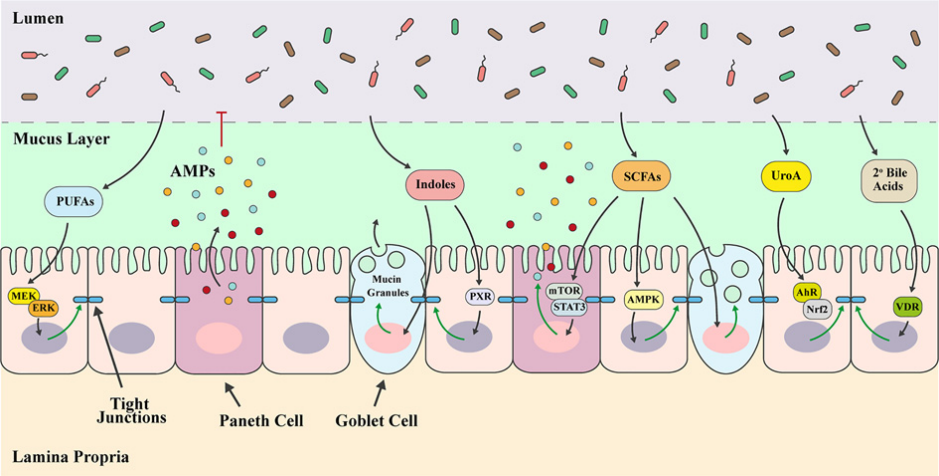
The direct effect of microbiota-derived metabolites on intestinal barrier integrity Microbiota-derived metabolites play an important role in maintaining intestinal barrier integrity to prevent epithelial damage and limit mucosal inflammation. Metabolites of polyunsaturated fatty acids (PUFAs) via a GPR40-MEK-ERK pathway have been shown to prevent loss of TJ proteins. Indoles, SCFAs, UroA, and secondary bile acids have also been shown to increase expression of TJ proteins via pathways involving PXR, adenosine monophosphate-activated protein kinase (AMPK), AhR-Nrf2, and VDR, respectively. Indoles and SCFAs promote the production and secretion of mucin, reinforcing the mucus layer. SCFAs activate a mechanistic target of rapamycin (mTOR)-signal transducer and activator of transcription (STAT) 3 pathway in a GPR43-dependent manner to induce production of AMPs.

Normal maturation and function of the mucus layer is strongly influenced by the gut microbiota, either through bacterial degradation and turn-over of mucin glycans or by bacteria-mediated processes to regulate host glycosylation of mucins [111]. Additionally, microbe-derived signals and metabolites have been shown to protect the intestinal epithelial barrier, up-regulating and strengthening cell junctions as well as promoting maintenance of the mucus layer and release of antimicrobial molecules (Figure 2). For example, indoles, which are microbiota-derived metabolites produced from the amino acid tryptophan have been shown to increase gene expression linked to TJ formation and mucus production [112,113]. Indoles further protect IECs through attenuation of tumour necrosis factor-α (TNF-α)-mediated activation of nuclear factor κ-light-chain-enhancer of activated B cells (NF-κB), decreased expression of pro-inflammatory cytokine interleukin (IL)-8, reduced attachment of pathogenic *E. coli*, and increased expression of anti-inflammatory IL-10 [112]. Studies using mice have shown that indole 3-propionic acid (IPA) stimulates the pregnane X receptor (PXR) resulting in up-regulation of TJ proteins in enterocytes and down-regulation of TNF-α [114]. Urolithin A (UroA), a sole microbiota-derived metabolite produced from polyphenolic compounds also enhances intestinal barrier integrity by increasing TJ proteins in IECs through activation of aryl hydrocarbon receptor (AhR)-nuclear factor erythroid 2-related factor 2 (Nrf2)-dependent pathways [115]. SCFAs, in particular butyrate, are the main energy source for colonocytes and are known to promote epithelial barrier integrity [116–119]. SCFAs are taken up by cells either by passive diffusion or facilitated by solute transporters such as monocarboxylate-transporter 1 (MCT-1) and sodium-coupled MCT-1 (SMCT1) where they can then be detected by intracellular receptors such as peroxisome proliferator-activated receptor γ (PPARγ) [120–122]. Alternatively, SCFAs may signal through G-protein coupled receptors (GPRs), such as GPR41, GPR43, and GPR109A, to activate signalling cascades that regulate immune responses [123–125]. SCFAs directly promote mucosal barrier integrity through induction of genes encoding TJ proteins [126], mucins [127], and AMPs [128]. The gut microbial-derived metabolite of polyunsaturated omega-6 fatty acid linoleic acid, 10-hydroxy-*cis*-12-octadecenoic acid (HYA), is able to ameliorate intestinal barrier damage and changes to cell junction proteins partially via a GPR40-mitogen activated protein kinase kinase (MEK)-extracellular signal-regulated kinase (ERK) pathway [129]. Secondary bile acids, such as lithocholic acid [48], produced by gut microbial conversion of primary bile acids, have also been shown to protect IECs from a TNF-α-induced decrease in TJ proteins through activation of the vitamin D receptor (VDR) [130].

### Mucosal immune regulation by the microbiota

The mucosal immune system is fundamental to intestinal barrier integrity and inflammation. The microbiota plays a vital role, especially in early life, in the maturation and regulation of host immunity to ensure mucosal inflammation is controlled and that the host can differentiate between commensal and pathogenic bacteria [131].

Commensal bacteria have long been associated with the correct development of mucosa-associated lymphoid tissues (MALT), in particular the gut-associated lymphoid tissue (GALT) which includes Peyer’s patches. Early studies using germ-free (GF) mice have shown that the absence of a commensal microbiota correlates with extensive defects in lymphoid tissue architecture and immune responses [132]. A significant reduction in intra-epithelial lymphocytes (IELs), such as αβ and γδ IELs, as well as secretory IgA, is seen in GF mice (compared with their colonised counterparts), which can be reversed following microbial colonisation [133,134]. Gestational maternal colonisation in mice has been shown to increase immune cell subtypes including intestinal group 3 innate lymphoid cells (ILC3s) and F4/80^+^ CD11c^+^ mononuclear cells [135]. Pro-inflammatory IL-17^+^ CD4^+^ T helper (Th17) cells, which normally exist in large numbers in the lamina propria of the small intestine are absent from GF mice, however, they can be induced upon commensal colonisation [136–138]. This is most notable with segmented filamentous bacteria (SFB), which upon adhesion to IECs, are known to stimulate T-cell responses as well as enhance IgA production [126,139]. PSA from *B. fragilis* aids cellular and physical maturation of the developing immune system in mice, correcting T-cell deficiencies and imbalances in CD4^+^ T helper 1 (Th1) and Th2 cell subtypes, directing lymphoid organogenesis [140]. In neonatal mice, *B. fragilis* is also known to supplement the endogenous lipid antigen milieu with inhibitory sphingolipids, impeding invariant natural killer T (iNKT) cell proliferation in the colonic lamina propria, providing protection against iNKT cell-mediated mucosal inflammation and injury [141]. Microbial colonisation also influences the development of early B-cell lineages in the intestinal mucosa, modulating gut immunoglobulin repertoires [142]. Sufficient intestinal microbiota diversity during early life colonisation has been shown to be essential for the establishment of an immunoregulatory network that protects against elevated induction of IgE at mucosal sites, which is linked to immune hypersensitivity, mucosal inflammation, and allergies [72].

**Figure 3:**
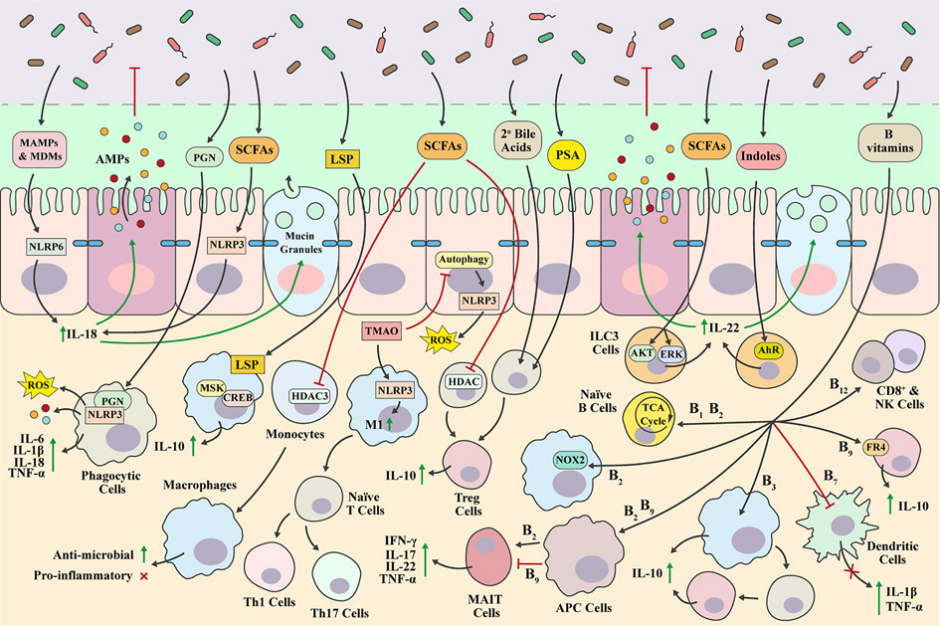
Regulation of mucosal immunity by the intestinal microbiota The mucosal immune system is complex with cross-talk between both innate and adaptive components that are primed to counter pathogens and preserve mucosal barrier integrity. MAMPs and microbial-derived metabolites (MDMs) can directly influence this network, aiding the development of host immune responses against pathogens whilst also limiting excessive mucosal inflammation to ensure microbiota tolerance.

Beyond infancy, the gut microbiota continues to influence the host immune system to maintain host–microbiota symbiosis and intestinal homoeostasis (Figure 3). For example, MAMPs and microbiota-derived metabolites can signal through activation of NLR complexes, called inflammasomes, to shape host immune responses and regulate mucosal barrier function. The microbiota induces NOD-, leucine-rich repeat (LRR)-, and pyrin domain containing 6 (NLRP6) inflammasome signalling to promote steady-state pro-inflammatory IL-18 mucosal secretion, which in turn activates AMP and mucin production in the intestinal mucosa, refining microbiota composition [143]. SCFAs signal through GPR43 and GPR109A to activate NLRP3 leading to IL-18 mucosal secretion [124]. Members of the microbiota, specifically *Proteus mirabilis*, can induce robust IL-1β production via the NLRP3 inflammasome to promote intestinal mucosal inflammation, mediated by monocytes that are recruited to the intestine in response to epithelial injury [144]. The sensing of PGN fragments and PGN from intact commensal bacteria through multiple PPRs is necessary for the proper development and activation of immune cells. Phagocytes sense internalised PGNs through NLRs and inflammasome complexes (e.g. NLRP3) which induce secretion of pro-inflammatory cytokines (e.g. TNF-α, IL-6, IL-1β, and IL-18) as well as increase antimicrobial responses, such as reactive oxygen species (ROS) and AMP production [145]. Macrophages play a vital role as innate immune effector cells to maintain intestinal homoeostasis, being able to initiate both pro-inflammatory and anti-inflammatory signalling pathways. In mice, intestinal microbial colonisation has been shown to drive continuous replenishment of macrophages in the intestinal mucosa by monocytes that express C–C chemokine receptor type 2 (CCR2) [146]. *Helicobacter hepaticus* induce an early IL-10 response in intestinal lamina propria-resident macrophages and produce a large soluble polysaccharide (LSP) that activates a specific mitogen and stress-activated protein kinase (MSK)/CREB-dependent anti-inflammatory signalling cascade via TLR2, aiding tolerance and mutualism [147]. Butyrate drives monocyte to macrophage differentiation through histone deacetylase (HDAC) 3 (HDAC3) inhibition to promote an antimicrobial state without inducing pro-inflammatory cytokine production [148]. Trimethylamine N-oxide (TMAO), the oxidated product of gut microbiota-derived trimethylamine, triggers M1 macrophage polarisation via NLRP3 inflammasome activation in mice resulting in Th1 and Th17 differentiation [149]. Furthermore, TMAO has been shown to prime the NLRP3 inflammasome and increase generation of ROS via inhibition of autophagy in colonic epithelial cells contributing to mucosal inflammation [150].

ILCs are a heterogeneous innate cell population that specialise in rapid secretion of polarising cytokines and are involved in the initiation of mucosal inflammation to fight infection and inflammatory resolution for mucosal tissue repair [151,152]. Many of the functions of ILCs are mediated by the microbiota [152,153]. For example, proliferation and function of colonic ILC3s is regulated by SCFA activation of GPR43. GPR43 agonism differentially activates protein kinase B (AKT) and ERK signalling, leading to increased colonic ILC3-derived IL-22, ensuring correct mucosal mucin and AMP production from IECs [154,155]. Dichotomous regulation of ILCs has been observed by a pair of *Helicobacter* species, activating ILCs but negatively regulating proliferation of ILC3s [156].

PSA mediates the conversion of CD4^+^ cells into anti-inflammatory forkhead box P3 (Foxp3)^+^ regulatory T (Treg) cells and subsequent production of IL-10, both via TLR2, to suppress mucosal inflammation [157]. SCFAs, such as butyrate and propionate, also induce Treg generation via HDAC inhibition [158]. Microbiota-derived secondary bile acids have recently been shown to regulate colonic retinoic acid receptor-related orphan receptor γ (RORγ)+ Treg induction and homoeostasis [159]. Indoles, such as indole-3-aldehyde, signal through AhR in immune cells to regulate IL-22 production and promote mucosal immune homoeostasis [160]. Bacteria-derived B vitamins have an impact on many aspects of immunological maintenance [161]. Vitamins B1 and B2 act as cofactors for enzymes involved in the TCA cycle and are important for immunometabolism and immune cell differentiation [161,162]. Vitamin B2 is also associated with ROS generation in phagocytic immune cells through priming nicotinamide adenine dinucleotide phosphate (NADPH) oxidase 2 (NOX2) [163]. The vitamin B2 metabolite, 6-hydroxymethyl-8-d-ribityllumazine, bound to major histocompatibility complex (MHC) class I-related protein (MR1) on antigen-presenting cells (APCs), activates mucosal-associated invariant T (MAIT) cells to promote production of pro-inflammatory interferon-γ (IFN-γ) and IL-17 [164]. In contrast, the vitamin B9 metabolite, acetyl-6-formylpterin, inhibits activation of MAIT cells [165]. Vitamin B3 binds to GPR109A on macrophages and dendritic cells leading to an increase in anti-inflammatory cytokines and Treg differentiation [166]. Vitamin B7 (biotin) suppresses the production of pro-inflammatory cytokines [167,168]. Vitamin B9 (folate) binds to the folate receptor 4 (FR4) on differentiated Tregs, promoting cell survival [161]. Vitamin B12 is required for CD8^+^ T cell differentiation and NK cell activation [169].

As detailed, the intestinal microbiota is not functionally independent from the host mucosa, playing an important role in gut homoeostasis. When there is a perturbation in this finely balanced relationship, loss of mucosal barrier integrity and a rise in abnormal immune responses can occur leading to a risk of sustained pathogenic inflammation and development of disease.

## Dysbiosis and disease

**Figure 4:**
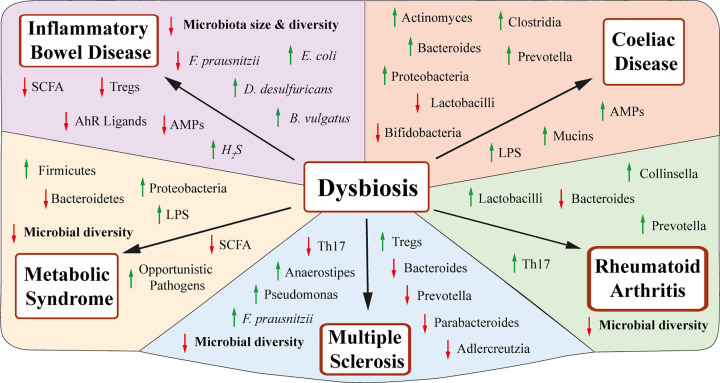
Linking dysbiosis and disease Several diseases have been linked to dysbiosis. A dysbiotic state is often characterised by a loss of beneficial microbes, increased levels of pathobionts, and a decrease in microbial diversity. Changes in relative bacterial abundance, as well as microbe-derived metabolites, are thought to cause dysregulation in host gut permeability, leading to a compromised immune response and in turn the development of disease.

Environmental changes as well as host genetic susceptibility can contribute to dysbiosis [170,171]. In a dysbiotic state, altered relative abundances of certain microbial species and/or microbiota-derived metabolites can lead to the disruption of intestinal barrier integrity and host immune responses. Dysregulated mucosal immune responses are often characterised by an up-regulation of Th1, Th2, and Th17 cells and a down-regulation of Tregs and IgA [172,173]. Dysbiosis is linked to the development of numerous disease states including IBD, rheumatoid arthritis (RA), multiple sclerosis (MS), and metabolic syndrome [172,174] (Figure 4). However, it is worth noting that many of the studies to date, particularly those highlighting immunological pathways, have been solely based on findings from rodent models, which have inherent limitations [175].

### IBD

IBD is an umbrella term encompassing a group of complex chronic inflammatory disorders of the gastrointestinal tract [176]. Most commonly in the form of Crohn’s disease (CD) and ulcerative colitis (UC), IBD has been associated with changes in gut microbiota. However, it is not clear whether these changes contribute to disease pathogenesis or develop because of disease-related inflammation. IBD patients exhibit a reduction in microbiota size, functional diversity, and stability compared with healthy controls. In general, the microbiome of IBD patients show a decrease in Firmicutes of the *Clostridium leptum* group, particularly *Faecalibacterium prausnitzii (F. prausnitzii)*, and an increase in Bacteroidetes and Proteobacteria such as *Desulfovibrio desulfuricans (D. desulfricans)* and *E. coli* [177–179]. On average, IBD patients harbour 25% less microbial genes than a healthy person [180].

The changes observed in the gut microbiome of IBD patients have been linked to bacteria known to have a role in either suppressing or promoting inflammation. Individuals with CD have a lower abundance of *F. prausnitzii*, an SCFA-producing bacterium, that promotes good gut health through up-regulation of Tregs and anti-inflammatory cytokines [181,182]. In humans, a reduction in *F. prausnitzii* is associated with an increased risk of postoperative recurrence of CD [182]. Furthermore, in IBD patients, an increase in the abundance of sulphate-reducing bacteria, such as *D. desulfuricans*, is attributed to increased production of hydrogen sulphate, which can damage IECs and in turn induce mucosal inflammation [183,184]. Several human studies have also reported a mucosa-associated *E. coli* richness in CD patients [179,185], leading to increased gut permeability and inflammation [186]. Both human and murine models have found that a reduction in tryptophan levels are also associated with IBD [187,188]. In IBD patients, tryptophan serum levels were found to inversely correlate with IL-22 levels and disease activity [187].

### Coeliac disease

Coeliac disease, prevalent in 1–2% of the global population, is an immune-mediated inflammatory disorder that primarily affects the small intestine and is initiated following ingestion of gluten in genetically predisposed individuals [189,190]. Research has suggested that dysbiosis plays a role in triggering coeliac disease with a dysregulated immune response and failure to maintain intestinal barrier integrity, leading to mucosal inflammation [191]. However, like IBD, it remains unclear as to whether the dysbiotic state characteristic of coeliac disease is a cause or consequence of a dysregulated immune response.

As coeliac disease generally presents in childhood and young adulthood, most studies looking at a link between coeliac disease and the microbiome have focused on children [191]. Rod-shaped bacteria, including Clostridia, Provotella, and Actinomyces, are more frequently found in the small bowel of children with active coeliac compared with healthy controls [192]. Whilst no consistent microbial signature has been determined for patients with coeliac disease, most studies report an imbalance between Gram-negative and Gram-positive bacteria, characterised by both an increase in Gram-negative Bacteroides and Proteobacteria, and a decrease in Gram-positive Lactobacilli and Bifidobacteria, which have a protective anti-inflammatory effect [193,194]. Experimental murine models have reported that some Bacteroidetes species are involved in the disruption of intestinal barrier integrity, exhibiting pro-inflammatory effects [46,195,196]. Both mice and human studies have shown that Lactobacilli and Bifidobacteria may play a role in modifying the immunogenic potential of gluten, through breakdown of both gluten and its peptide derivatives [197,198]. For example, Lactobacilli can detoxify gliadin peptides after their partial digestion by human proteases. Both mice and human studies report that *Bifidobacterium* strains also play a role in reducing the epithelial permeability triggered by gluten, diminishing pro-inflammatory cytokine synthesis and decreasing jejunal barrier damage [199–201].

Whilst the exact mechanisms involved in coeliac disease remain unclear, studies in mice have shown that a dysbiotic microbiota can result in increased levels of LPS in the intestine, which result in a dysregulation of the immune response through the activation of both IELs and IECs, triggering the production of AMPs and mucin [202,203]. Additionally, mouse studies have linked alterations in microbial metabolites to the induction of Treg cells and dendritic cells, which produce IL-10 and retinoic acid and thereby contribute to the activation of various cellular inflammatory processes within the lamina propria [158,204].

### Other autoimmune diseases

RA is a systemic autoimmune disorder that results in joint destruction, affecting approximately 0.5–1% of the global population [205]. Patients with RA exhibit decreased gut microbial diversity and microbial gut dysbiosis characterised by an abundance of *Prevotella*, Lactobacilli, and Collinsella [206–208]. Mouse models show that *Prevotella* and *Collinsella* can induce a pro-inflammatory Th17 response and increase gut permeability [206]. Colonisation of K/BxN mice, an established RA model, with SFB was shown to induce Th17 cell proliferation, ultimately leading to the differentiation of B cells and the production of autoantibodies [209]. It is thought that these autoantibodies target joints, leading to the inflammation seen in RA.

MS is a neurodegenerative autoimmune disease that affects the central nervous system (CNS) [210]. Whilst a typical microbiota phenotype for MS has not yet been described, patients with active disease generally exhibit decreased species richness, an abundance of *Anaerostipes, Faecalibacteria*, and *Psuedomonas*, and decreased levels of Bacteroides, Prevotella, Parabacteroides and Adlercreutzia [211,212]. An autoimmune encephalomyelitis (EAE) GF mouse model, which is also a model for MS, showed lower levels of IL-17 in both the gut and CNS, and an increase in peripheral Tregs [213]. Furthermore, disease severity in EAE models is also closely related to altered intestinal permeability, reduced submucosa thickness, and altered TJ expression in IECs [214,215].

### Metabolic syndrome

Metabolic syndrome describes a group of risk factors, including obesity, hyperglycaemia, hypertension, and dyslipidaemia, which can lead to the development of various conditions including cardiovascular disease. The pathogenesis of metabolic syndrome is linked to a variety of factors such as insulin resistance, chronic low-grade inflammation in metabolic tissue, and oxidative stress [216]. In recent years, gut dysbiosis has been identified as a risk factor for metabolic syndrome [217]. It is believed that environmental factors, such as a high-fat diet, linked to decreased microbial diversity, promotes both general and metabolic tissue inflammation that may lead to the development of metabolic syndrome.

The links between the gut microbiota and both obesity and type 2 diabetes (T2D) have been most extensively studied. Studies in mice have shown that transplantation of gut microbiota from obese to lean GF mice resulted in an obesogenic phenotype [218]. Furthermore, GF mice fed a high-fat, high-sugar diet were found to be resistant to weight gain [219]. Studies in humans suggest that compared with lean individuals, obese individuals have increased levels of Firmicutes and decreased levels of Bacteroidetes [220,221]. In both humans and murine models, Roux-en-Y gastric bypass surgery has been found to rapidly change the gut microbiota, with gut microbiota normalising close to non-obese controls [222,223]. Patients with T2D typically exhibit reduced microbiome diversity, reduced SCFA-producing bacteria, and an increased number of opportunistic pathogens [224]. Rodent studies have found that SCFA play a key role in metabolic disorders, particularly in obesity and T2D. The SCFAs, propionate and acetate, were found to influence gut motility, intestinal transit rate, and caloric energy extraction from the diet through GPR41 activation [225]. Increased insulin sensitivity and increased satiety was also observed in mouse models, thought to be linked to the induction of glucagon-like peptide (GLP)-1 secretion through the activation of GPR43 and GPR41 [226]. Butyrate provides energy to enterocytes by exerting a trophic effect and inducing GLP-2 synthesis that in turn strengthens the gut barrier function [227].

It has been suggested that the gut microbial dysbiosis experienced in metabolic disorders leads to impaired intestinal cell function and increased gut permeability, partly induced by a high-fat diet [228]. Rodent studies have reported an increase in Gram-negative bacteria, including Proteobacteria, leading to a local increase in LPS in the mucosal layer [229,230]. MAMPs and microbiota-derived metabolites, including LPS, can translocate through the epithelial layer and reach the lamina propria where they are internalised by phagocytes. Furthermore, it has been hypothesised that microbial gut dysbiosis impairs communication between phagocytes and other immune cells in animal models, allowing the translocation of bacterial components to metabolic tissue [231,232]. In the metabolic tissue of mice, bacterial components trigger inflammation by promoting the proliferation of preadipocytes and macrophages, increasing ILC3 frequency and increasing the infiltration of B and T lymphocytes. Associated pro-inflammatory cytokines can also contribute to reduced insulin signalling, exacerbating the effects of diabetes.

## Microbiota-targeted therapies

As detailed previously, dysbiosis in the gut is implicated in multiple gastrointestinal and non-gastrointestinal diseases. Intervention aiming to ameliorate this pathological environment with the delivery of targeted beneficial or wholesale bacterial populations in the form of probiotics and faecal microbiota transplantation (FMT), respectively, has been in clinical practice for many years [233,234]. The various mechanisms by which probiotics and FMT exert their therapeutic effect has been reviewed in detail elsewhere [235–237], but centre their interaction with the host mucosal immune system via MAMPs [238,239] or extracellular vesicles [240–242], the surrounding microbiota via AMPs [243], microbial cross-feeding [244] or nutrient competition, and their contribution to the broader mucosal metabolic environment [118,148]. Here we review the latest developments and innovations in probiotics and FMT.

### Probiotics

The main probiotic genera, including Lactobacilli, Bifidobacteria, Saccharomyces, and Streptococci, as well as combination commercial probiotics, have been researched extensively and touted as potential therapies for many diseases or symptoms [245]. Certain probiotic strains have discrete effects on mucosal immune function, such as that seen with *Lactobacillus plantarum* TIFN1010 which modulates gene transcription pathways related to cell-cell adhesion and mucosal healing processes [238]. However, robust clinical data to support their use remain limited, with systematic reviews in IBD [246–248], Irritable bowel syndrome (IBS) [249] and *C. difficile*-associated diarrhoea (CDAD) [250] showing neutral or only qualified evidence for use. Similarly, practice guidelines do not recommend the routine use of probiotics [233], partly due to uncertainty regarding species or strain-dependent effects [251,252].

Recent advances in genomic sequencing and metabolic modelling have offered a way to reduce microbial uncertainty and the chance to optimise probiotic use through genetic engineering tools such as CRISPR-Cas [253–256]. For example, genetic modification of *Lactobacillus casei* (*L. casei*) to overexpress the *mcra* gene, and so enhance bioactive compound production, such as conjugate linoleic acid, can result in elimination *of Campylobacter jejuni*, an important diarrhoea-associated pathogen [257]. As well as optimising established probiotic species such as *L. casei*, confirmation of species such as *Akkermansia muciniphilia* (*A. muciniphilia*) as probiotic therapeutic candidates has become possible through the use of genome-scale modelling. Using this method, the complete microbial genome sequence can be screened to predict genes that influence particular metabolic pathways [258]. For *A. muciniphilia*, genes linked to sugar degradation and vitamin biosynthesis, as well as SCFA production, were predicted using this approach and validated by transcriptomic and proteomic analysis *in vitro* [259]. Antibiotic resistance and metabolic variation can also be assessed by whole-genome assembly undertaken on patient-derived stool samples [260]. Genomic sequencing technology has also been used to identify individuals resistant to probiotic colonisation at the mucosal level [261], allowing therapy to then be tailored, reducing treatment variability currently seen with probiotics [262].

Overall, whilst significant advances in probiotic therapy have been made, there is a need for a greater understanding of probiotic formulation, in addition to a requirement for more robust human clinical trial data to justify its routine use.

### Postbiotics

An important additional consideration regarding probiotic preparations is the intrinsic effect of microbial cell surface components and metabolites. Whereas, by definition, probiotics are live microorganisms [263], there is also a role for postbiotics, as inanimate microorganisms and/or their components [264], prepared specifically for their health benefits on the host. These narrow criteria exclude purified microbial metabolites applied in isolation and instead focus on thermal inactivation and quantification of products that possess microbial effector molecules such as bile salt hydrolase [265] and exopolysaccharides [239].

Murine studies have shown the effect of postbiotics on gastrointestinal mucosa in a *Citrobacter*-induced colitis model [266], whereas the mechanistic impact on mucosal inflammation in humans is more limited to specific metabolites such as butyrate as in the case of diversion colitis [267]. However, clinical studies focussing on subjective outcomes such as symptom scores have shown benefit of postbiotics in IBS [268].

To date, the application of postbiotics in gastrointestinal disease remains limited with the mainstay of evidence [269,270] and regulation [271], centred on secondary prevention of respiratory infections. Further mechanistic and clinical trial data are required to characterise the effect of specific postbiotics on gastrointestinal inflammation.

### FMT

FMT, the delivery of donor stool into the gastrointestinal tract of a patient, is an established and guideline-supported intervention for rCDI [234,272,273], independent of route of delivery [274], and a potential option in severe primary CDI [275]. Meta-analysis has indicated a positive association between FMT and the treatment of IBD, particularly with active UC [276,277]. Further trials are currently underway [278] to confirm FMT efficacy before being adopted into routine clinical practice [279]. Similarly, with CD, there is evidence supporting the benefits of FMT [280], however, it has not yet been recommended for clinical use [281]. Evidence remains lacking for routine use of FMT in IBS [282] with evidence for only conditional use in metabolic syndrome [283,284] and hepatic encephalopathy [285]. The use of FMT in non-gastrointestinal diseases is an area of ongoing study with randomised clinical trials in type 1 diabetes showing promise [286]. FMT clinicals trials are also underway to assess effectivity in treating Coeliac disease (NCT 04014413), RA (NCT03944096), Sjogren’s syndrome (NCT03926286) and MS (NCT03183869; NCT03975413; NCT04150549), building upon prior animal and uncontrolled human studies [287]. It is in malignancy, and specifically anticancer immunotherapies, where microbiota and their manipulation have shown great promise, building on evidence that certain genera, for example, Bifidobacteria [288] or Bacteroides [289], can affect the efficacy of malignant melanoma treatments. A recent clinical trial revealed that some patients refractory to anti-programmed cell death protein 1 (PD-1) immunotherapy could overcome this resistance to therapy by undergoing FMT from donors who were responders to the same anti-PD-1 immunotherapy [290]. PD-1 is an immune checkpoint receptor on T cells that prevents overstimulation of immune responses and contributes to the maintenance of immune tolerance to self-antigens. The fact that FMT impacts on anti-PD-1 melanoma therapy demonstrates that the composition of the microbiota influences host systemic immune responses. FMT is now being applied to metastatic hormone-resistant prostate cancer (NCT04116775) and to ameliorate chemotherapy-induced toxicity (NCT04040712).

An important factor in FMT is the role of viruses and mycobiota given that whole stool transplantation involves a transfer of these microorganisms to the new host along with bacteria. Bacteriophages contribute to host immunity by adhering to mucosal mucus creating an additional antimicrobial layer that reduces bacterial attachment and colonisation of the mucosa [291]. Both Caudovirales [292] and *Saccharomyces* [293] have been shown as important drivers for successful treatment of rCDI by FMT. Faecal filtrate transfer (FFT), a supernatant composed of bacterial debris, AMPs, metabolic products and oligonucleotides, but not live bacteria, was also seen to improve outcomes in rCDI [294].

A limitation of widespread FMT use outside of the trial setting is the conceptual acceptability of single or pooled donor stool being transferred to a patient. Synthetic microbiomes can be cultured from donors and purified, or compiled from metagenomic studies [295]. Purified intestinal bacterial culture have been shown to be as effective in treating rCDI in a proof-of-principle study [296]. A recent randomised-controlled trial reported that a 12-strain bacterial mixture cultured from donor stool was inferior to conventional FMT but equivalent to using vancomycin for the treatment of rCDI [297]. FMT using freeze-dried or lyophilised matter has been shown in observational studies to also be effective in treating rCDI [298], with a propagated, lyophilised and encapsulated formulation currently under investigation in clinical trials for the treatment of rCDI (NCT02865616), UC (NCT03832400), and other diseases. These technologies, if efficacy is confirmed, herald the opportunity of a ‘post-FMT’ treatment model centred on highly selected donors yielding a purified, standardised, and cryopreserved microbiota preparation for systematic clinical use.

## Conclusion

The microbiome is a metabolically and immunologically active presence within the gastrointestinal tract that plays a vital role in the maintenance of human health. This population of highly diverse microorganisms is shaped by numerous factors, most notably, diet and the use of medications such as antibiotics. The intestinal mucosa provides an important interface between the microbiota and host, where the microbiota not only aids in development of effective host immune responses against pathogens and injury but also limits excessive mucosal inflammation to promote tolerance and stability of the gut environment. Microbial components and microbe-derived metabolites contribute to both mucosal barrier integrity and the regulation of underlying immune responses to preserve intestinal homoeostasis. When there is a loss of this balanced relationship, as seen in dysbiosis, then there is a risk of sustained pathogenic inflammation and the development of numerous diseases. As our understanding of the microbiome and microbiota–host interactions has improved, so has our ability to harness members of the microbiota to reverse dysbiosis, reduce mucosal inflammation, and prevent disease progression. The outcome of ongoing clinical trials and mechanistic studies will hopefully extend our current knowledge of the microbiome and further our understanding of the role it plays in mucosal inflammation.

## Open Access

Open access for this article was enabled by the participation of University College London in an all-inclusive *Read & Publish* pilot with Portland Press and the Biochemical Society under a transformative agreement with JISC.

## Abbreviations

AhR: aryl hydrocarbon receptor
AMP: antimicrobial peptide
CD: Crohn’s disease
CNS: central nervous system
CREB: cAMP response element-binding protein
C-section: caesarean section
EAE: autoimmune encephalomyelitis
ERK: extracellular signal-regulated kinase
FMT: faecal microbiota transplantation
GF: germ-free
GLP: glucagon-like peptide
GPR: G-protein coupled receptor
HDAC: histone deacetylase
IBD: inflammatory bowel disease
IBS: irritable bowel syndrome
IEC: intestinal epithelial cell
IEL: intra-epithelial lymphocyte
IL: interleukin
ILC: innate lymphoid cell
ILC3: intestinal group 3 innate lymphoid cell
LPS: lipopolysaccharide
MAIT: mucosal-associated invariant T
MAMP: microbe-associated molecular pattern
MS: multiple sclerosis
NLR: nucleotide-binding oligomerisation domain (NOD)-like receptor
NLRP: NOD-, leucine-rich repeat (LRR)-, and pyrin domain containing
NOD: nucleotide-binding oligomerisation domain
PD-1: programmed cell death protein 1
PGN: peptidoglycan
PPI: proton pump inhibitor
PRR: pattern-recognition receptor
PSA: polysaccharide A
RA: rheumatoid arthritis
rCDI: recurrent *Clostridium difficile* infection
ROS: reactive oxygen species
SCFA: short-chain fatty acid
Th: T helper
TJ: tight junction
TLR: Toll-like receptor
TMAO: trimethylamine N-oxide
TNF-α: tumour necrosis factor-α
Treg: regulatory T
T2D: type 2 diabetes
UC: ulcerative colitis
